# Synergistic Multisystem Photocatalytic Degradation of Anionic and Cationic Dyes Using Graphitic Phase Carbon Nitride

**DOI:** 10.3390/molecules28062796

**Published:** 2023-03-20

**Authors:** Wen Yang, Kun Ding, Guangzhou Chen, Hua Wang, Xinyue Deng

**Affiliations:** 1 College of Environmental and Energy Engineering, Anhui Jianzhu University, Hefei 230601, China; 2 Anhui Key Laboratory of Environmental Pollution Control and Waste Resource Utilization, Anhui Jianzhu University, Hefei 230601, China

**Keywords:** g-C_3_N_4_, photocatalysis, synergistic multisystem, anionic and cationic dyes, dynamics, zeta potential

## Abstract

Graphitic phase carbon nitride (g-C_3_N_4_) is a promising photocatalytic environmental material. For this study, the graphitic phase carbon nitride was prepared using a thermal polymerization method. The characteristic peaks, structures, and morphologies were determined using Fourier-transform infrared spectroscopy (FT-IR), X-ray diffractometry (XRD), and scanning electron microscopy (SEM), respectively. Under the synergetic visible light catalysis of H_2_O_2_ and Na_2_S_2_O_8_, the degradation effects of g-C_3_N_4_ on the anionic dye methyl orange (MO) and the cationic dye rhodamine b (Rhb) were investigated. The effects of adding different volumes of H_2_O_2_ and Na_2_S_2_O_8_ were likewise tested. The results showed that the above two synergistic systems increased the degradation rates of MO and Rhb by 2.5 and 3.5 times, respectively, compared with pure g-C_3_N_4_, and that the degradation rates of both MO and Rhb reached 100% within 120 min and 90 min, respectively, in accordance with the primary reaction kinetics. When H_2_O_2_ and Na_2_S_2_O_8_ were added dropwise at 10 mL each, the degradation rates of MO and Rhb were 82.22% and 99.81%, respectively, after 30 min of open light. The results of experiments upon both zeta potential and radical quenching showed that ·OH and ·O_2_^−^ were the main active radicals for dye degradation in our synergistic system. In addition, stability tests showed that the photocatalysts in the synergistic system still had good reusability. Therefore, the use of a synergistic system can effectively reduce the photogenerated electron-hole pair complexation rate, representing a significant improvement in both photocatalytic degradation and for stability levels.

## 1. Introduction

With the rapid developments in industrialization, water pollution has become one of the main problems affecting people’s everyday lives [1,2,3,4]. Among them, dye wastewater is an important part of water pollution [5,6,7]. Dyes are widely used in various industries such as textiles, leather, and paper, and it has been reported that about 30% of synthetic dyes are discharged into the environment [8,9,10]. Dye wastewater is complex and difficult to degrade, contains chromogenic and polar groups, and can cause eutrophication of water bodies [11,12,13]. Dyes can induce allergies and cancer, damage the respiratory system, nervous system and skin, and cause harm to the human body. Therefore, finding an effective and environmentally friendly method to degrade dyestuffs has become an urgent problem [14].

Photocatalytic methods, being one of the more advanced oxidation techniques, can oxidize pollutants into harmless small molecules, such as H_2_O and CO_2_, using free radicals generated during their reaction with light [15,16,17]. Photocatalytic technology can be applied to a wide range of pollutants with a wide range of situations. Lv et al. [18] used photocatalytic technology to degrade common air pollutants, with removal rates above 80%. Zeng et al. [19] used photocatalytic technology to treat low concentration ammonia nitrogen wastewater, with removal rates above 90%. TiO_2_ photocatalytic materials were discovered early on, but they have a large band gap in energy, and can only produce carrier electron-hole pairs in the UV range, its solar energy utilization being only about 4% [20,21]. Similarly, ZnO has a relatively wide band gap value (3.37 eV) and has a photocatalytic response only under UV light [22]. People have been looking for photocatalytic materials that can accept visible light. In recent years, graphitic phase carbon nitride (g-C_3_N_4_), with its two-dimensional layered structure, has been widely used in photocatalysis because of the narrow width of its forbidden band (about 2.7 eV), its high chemical stability, non-toxicity, and low preparation cost [23,24,25,26,27,28]. Saeed et al. [29] used photocatalysis as an effective tool to study dye degradation, and Zhou et al. [30] prepared g-C_3_N_4_-based photocatalytic concrete, which degraded 80% of their methylene blue within 30 min. Pandey et al. [31] removed 96% and 93% of PNP and Rhb dyes, respectively, using photocatalysis.

Dyes can be classified into cationic dyes, anionic dyes, and nonionic dyes [32]. The more common cationic dyes include rhodamine b (Rhb), malachite green, and methylene blue (MB), and common anionic dyes include methyl orange (MO) and Congo red [33,34]. Ivanenko et al. [35] achieved a good photocatalytic effect by degrading anionic and cationic dyes with ZnO synthesized using precipitation, but this method uses only 4% of the available sunlight in using only UV light, which is not a good use of sunlight and is therefore of no practical use. Gupta et al. [36] doped ZnSe with Cu under visible light conditions and cooperated with H_2_O_2_ to improve the degradation of methylene blue, where 15% Cu doping was shown to be able to achieve 98.09% MB degradation in 180 min; however, this photocatalytic material is complicated to prepare, and has some dangerous qualities and a long degradation time. The photocatalyst used in this study is simple to prepare, has high yield, and can degrade 100% of Rhb in 90 min and 100% of MO in 150 min using a synergistic reaction. At present, there are comparatively few studies regarding the use of g-C_3_N_4_ for the photocatalytic degradation of cationic and anionic dyes, and synergistic photocatalysis can greatly improve the reaction rate and degradation rate based on dyes in materials. Lu et al. [37] have achieved better results in the synergistic amine photocatalytic synthesis of imines using carbon-coated Pd/TiO_2_, which can exhibit excellent imine selectivity. Under visible light irradiation, H_2_O_2_ can be excited using light energy or electrons to produce superoxide radicals (OH), and Na_2_S_2_O_8_ can be excited using electrons to produce sulfate radicals (SO_4_^−^·) [38,39]. Thus, hydrogen peroxide (H_2_O_2_) and sodium persulfate (Na_2_S_2_O_8_) can be used for synergistic photocatalytic reactions. Su et al. [40] treated 2,4-dinitroanisole (DNAN) with ultraviolet (UV) photolysis combined with hydrogen peroxide (H_2_O_2_) oxidation. H_2_O_2_ can be adsorbed on active sites and then activate these sites. Liu et al. [41] used H_2_O_2_ as a green oxidant because its by-product is H_2_O. Methanol was then oxidized into methylformate (MF) using H_2_O_2_ oxidation.

In this study, g-C_3_N_4_ was prepared with a thermal polymerization method of simulating sunlight, and its characteristic peaks, structure, and morphology were characterized using Fourier-transform infrared spectroscopy (FT-IR), an X-ray diffractometer (XRD), and scanning electron microscopy (SEM). The photocatalytic effects of g-C_3_N_4_ on the cationic dye Rhb and the anionic dye MO were also investigated in concert with H_2_O_2_ and Na_2_S_2_O_8_ oxidants, both in accordance with the primary reaction kinetics. The degradation mechanism of the different systems was inferred from the quenching experiments of free radicals and the detection of zeta potential, and it was found that the main free radicals of the anionic dyes in the different synergistic systems were exactly opposite, which might be related to the acidity and basicity of the dyes themselves, which are yet to be explored.

## 2. Results and Discussion

### 2.1. Structural Characterization

#### 2.1.1. XRD and FTIR Analysis

**Figure 1:**
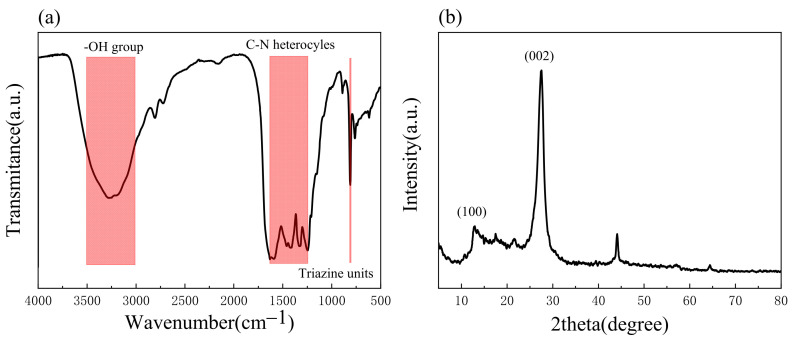
Infrared spectra and X-ray energy spectra of g-C_3_N_4_: (**a**) FT-IR; (**b**) XRD.

Figure 1a shows the FT-IR spectrum of g-C_3_N_4_. The surface functional groups of the prepared materials have been highlighted. The absorption peak at 809 cm^−1^ is caused by the deformation vibration of the N-C=N bond in the triazine ring, the absorption peak within the wave number from 1245 to 1631 cm^−1^ is due to the stretching vibration of the C-N bond, and the broad peak at 3000–3500 cm^−1^ is due to the stretching vibration of the -OH group on the surface of g-C_3_N_4_ [42,43,44,45].

As shown in Figure 1b, the crystal structure of g-C_3_N_4_ was determined using XRD, from which the characteristic peaks of the sample at 13.10 °C and 27.55 °C can be seen, where 13.10 °C and 27.55 °C correspond to the (100) and (002) crystal planes, respectively. The first diffraction peak is due to stacking within the triazine structural layer of the repeating unit in the plane, and the second diffraction peak is due to the interlayer stacking of the conjugated aromatic system that results from this [46,47]. This is a characteristic peak unique to the graphite structure, indicating the successful preparation of g-C_3_N_4_ [48,49].

#### 2.1.2. SEM Analysis

**Figure 2:**
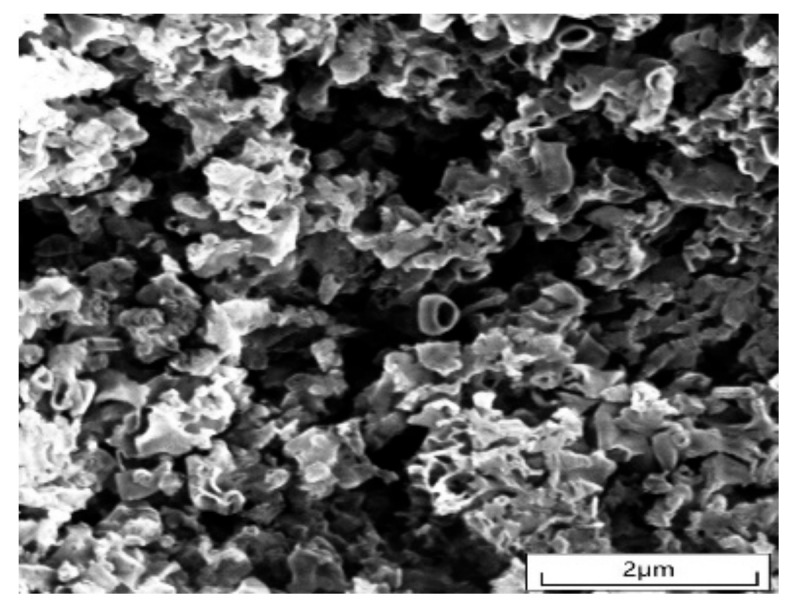
SEM image of g-C_3_N_4_.

Figure 2 shows the SEM image of g-C_3_N_4_, showing its change in morphology from layered stacking into an irregular, porous, tubular shape; this coral-like fluffy pore structure increases the specific surface area of the photocatalyst and provides more active sites for degrading pollutants, which can improve the degradation rate of photocatalysis to some extent [50,51,52,53].

### 2.2. Evaluation of the Degradation Performance of Dyes

**Figure 3:**
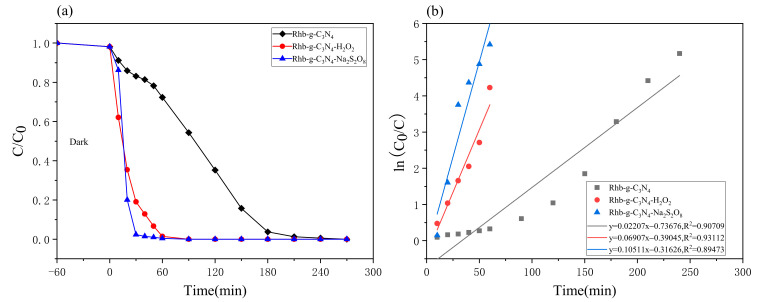
Rhb photocatalytic performance curves of different systems (photocatalyst dosage = 0.1 g, target pollutant = 50 mg/L): (**a**) photocatalytic activity of Rhb degradation photocatalyst under visible light irradiation; (**b**) the first-order kinetic curve of Rhb degradation photocatalyst under visible light irradiation.

**Figure 4:**
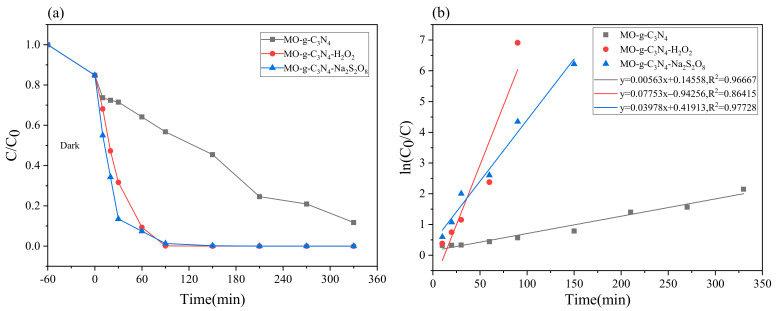
MO photocatalytic performance curves of different systems (photocatalyst dosage = 0.05 g, target pollutant = 20 mg/L): (**a**) photocatalytic activity of MO degradation photocatalyst under visible light irradiation; (**b**) the first-order kinetic curve of MO degradation photocatalyst under visible light irradiation.

In order to study the degradation performance of cationic and anionic dyes under two systems of H_2_O_2_ and Na_2_S_2_O_8_, g-C_3_N_4_ was added to 150 mL Rhb and 100 mL MO aqueous solutions, respectively, and then a certain amount of H_2_O_2_ and Na_2_S_2_O_8_ was added dropwise. Control experiments were conducted under LED, and the results are shown in Figure 3 and Figure 4.

As shown in Figure 3a, the concentration of Rhb solution decreased to a certain extent within 60 min of reaction with darkness, which was due to the adsorption of g-C_3_N_4_ itself. The degradation rate from pure g-C_3_N_4_ on the Rhb solution was 45.66% when the adsorption-desorption equilibrium was reached after direct light exposure for 90 min. In contrast, the removal effect of Rhb was significantly improved by adding H_2_O_2_ and Na_2_S_2_O_8_, respectively, and both reached a 100% degradation rate, a result which was 2.19 times higher than that of using pure g-C_3_N_4_ (see Table 1). This indicates that each of the two synergistic systems, H_2_O_2_ and Na_2_S_2_O_8_, played an important role in the photocatalytic degradation process of Rhb solution using g-C_3_N_4_, both in terms of reaction rate and degradation rate. Among them, the Na_2_S_2_O_8_ synergistic system showed somewhat higher speeds of degradation in the Rhb solution. In order to better observe the degradation pattern, the first-order reaction kinetics were fitted with experimental data according to Equation (11), and the results are shown in Figure 3b. The degradation rate of pure g-C_3_N_4_ was the slowest, at 0.02207 min^−1^; the degradation rate of H_2_O_2_ synergistic system was 0.06907 min^−1^; the highest degradation rate, that of the Na_2_S_2_O_8_ synergistic system, was 0.10511 min^−1^.

As shown in Figure 4a, the degradation rate of MO solution reached 15.18% after 60 min of adsorption-desorption, which is likewise due to the adsorption of g-C_3_N_4_ itself. After 90 min of exposure to open light, the degradation rates of the pure g-C_3_N_4_, H_2_O_2_, and Na_2_S_2_O_8_ synergistic systems reached 43.26%, 99.90%, and 98.70%, respectively (see Table 2). It is obvious that both g-C_3_N_4_ synergistic H_2_O_2_ and Na_2_S_2_O_8_ systems had a great degradation ability for MO solutions; among them all, g-C_3_N_4_ synergistic H_2_O_2_ system had higher degradation rate and reaction rate. As shown in Figure 4b, the MO photocatalytic process was consistent with quasi primary kinetics, and the degradation rates of pure g-C_3_N_4_, H_2_O_2_, and Na_2_S_2_O_8_ synergistic systems were k_1_ = 0.00563 min^−1^, k_2_ = 0.07753 min^−1^, and k_3_ = 0.03978 min^−1^, respectively.

**Figure 5:**
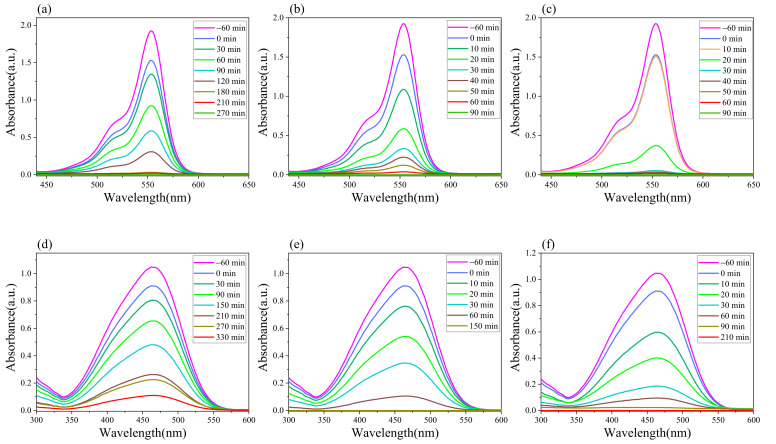
The absorption spectra of Rhb and MO under different catalytic conditions: (**a**) Rhb (g-C_3_N_4_); (**b**) Rhb (g-C_3_N_4_/H_2_O_2_); (**c**) Rhb (g-C_3_N_4_/Na_2_S_2_O_8_); (**d**) MO (g-C_3_N_4_); (**e**) MO (g-C_3_N_4_/H_2_O_2_); (**f**) MO (g-C_3_N_4_/Na_2_S_2_O_8_).

In order to observe the degradation of dyes in each synergistic system, the absorption spectra of Rhb and MO under different catalytic conditions were measured using an UV spectrophotometer at various times during the experiment, with the results shown in Figure 5. Under the synergistic systems, Rhb was completely degraded within 90 min, while MO was completely degraded within 150 min and 210 min, respectively. After reviewing the literature summaries for comparison, it was found that the photocatalyst in this study is comparatively simple to prepare and can be mass produced [54,55,56,57,58,59]. With synergistic photocatalysis, 100% of the dye can be degraded in a relatively short time, as shown in Table 3.

### 2.3. Effect of Volume Content

#### 2.3.1. Effect of Na_2_S_2_O_8_ Levels on Rhb Degradation

**Figure 6:**
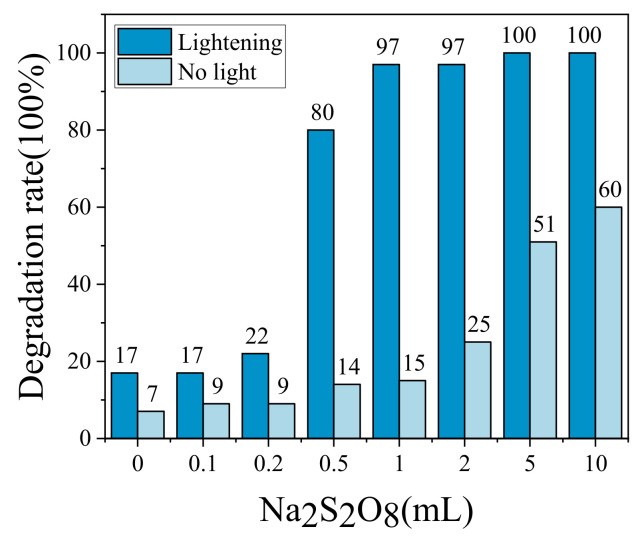
Effect of different amounts of Na_2_S_2_O_8_ on the degradation rate of Rhb.

The g-C_3_N_4_ synergistic Na_2_S_2_O_8_ system has higher speeds for Rhb solutions, so the photocatalytic degradation of the Rhb solution across different volumes of Na_2_S_2_O_8_ was investigated. Different volumes of Na_2_S_2_O_8_ were added dropwise to each solution at the end of the dark reaction, and the fixed light time across all those exposed to light was 30 min, with no light being used in the control experiment. The results of this are shown in Figure 6.

In the absence of light, the degradation rate increased with the increase in the amount of Na_2_S_2_O_8_ added dropwise. As can be seen from Figure 6, the Rhb degradation rate was only 7% without the addition of Na_2_S_2_O_8_; when 10 mL Na_2_S_2_O_8_ was added dropwise, the Rhb degradation rate increased by about 53% compared with the solution without Na_2_S_2_O_8_.

In the presence of light, the larger the volume of Na_2_S_2_O_8_, the better the synergistic photocatalytic effect. When Na_2_S_2_O_8_ was not added, the degradation rate was 17%. When the dropwise amount was greater than 0.5 mL, it began to increase rapidly, and the degradation rate increased from 22% to 80%, indicating that when Na_2_S_2_O_8_ was added dropwise to a certain amount, it also greatly promoted the redox reactions. When 10 mL of Na_2_S_2_O_8_ was added dropwise, the Rhb was basically completely degraded, with a degradation rate of 100%, which was 40% higher at this time compared with not having been exposed to light.

#### 2.3.2. Effect of H_2_O_2_ Volume on MO Degradation

**Figure 7:**
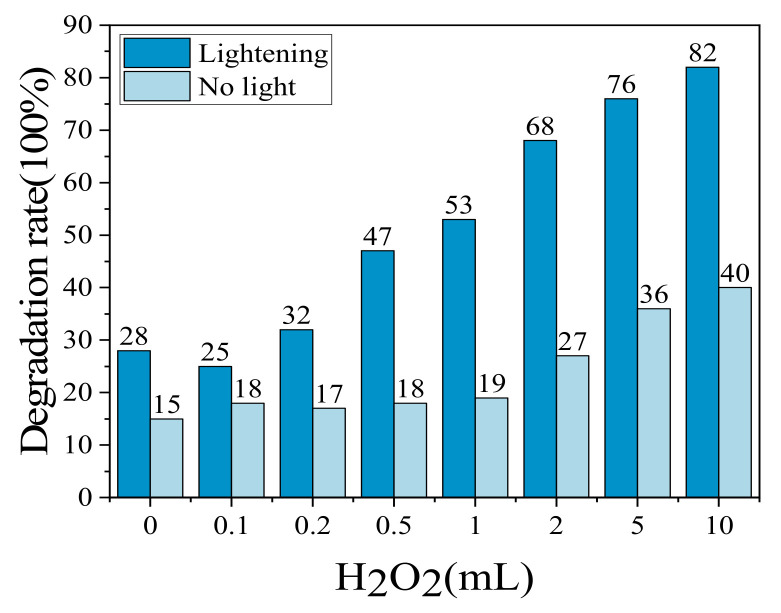
Effect of different amounts of H_2_O_2_ on the degradation rate of MO.

For the MO solution, H_2_O_2_ synergistic system has better photocatalytic effect, so it is important to explore the MO degradation rate under different H_2_O_2_ volume conditions. Different amounts of H_2_O_2_ were added dropwise at the end of the dark reaction for 60 min, respectively, and the light time was fixed to be 30 min for both. The light time was changed for the dark reaction, to serve as a control. The results are shown in Figure 7.

In the absence of light, the MO degradation rate was 15% when H_2_O_2_ was not added dropwise. However, the MO degradation rate decreased when the volume was increased from 0.1 mL to 0.2 mL (degradation rate changed from 18% to 17%), which was due to the H_2_O_2_ catalytically generated during the addition of 0.1 mL; the degradation rate increased starting from the increase to 2 mL (with a resulting degradation rate of 27%).

The degradation rates were all significantly increased when there was light, but when the amount of H_2_O_2_ added dropwise was 0.1 mL, the degradation of MO solution was inhibited to some extent. It is possible that a small amount of H_2_O_2_ was adsorbed onto the surface of g-C_3_N_4_ and occupied a certain spot. When 0.2 mL of H_2_O_2_ was added, the degradation rate of MO solution gradually increased (degradation rate of 32%); when 10 mL of H_2_O_2_ was added dropwise, the degradation rate of MO was 82%.

### 2.4. Investigation of the Mechanism of Different Systems

There are many studies on the photocatalytic degradation of Rhb and MO solutions using g-C_3_N_4_. After turning on the light source, g-C_3_N_4_ is induced to produce electrons (e^−^) and holes (h^+^) as in Equation (1). H_2_O is then oxidized with holes to produce ·OH and O_2_, as shown in Equations (2) and (3). O_2_ will be reduced with electrons to produce ·O_2_^−^, as in Equation (4). The generated reactive radicals (·OH, ·O_2_^−^) and cavities will degrade Rhb and MO solutions, as shown in Equation (5) [60,61,62].

(1) $${\mathrm{g-C}}_{3}N_{4}+\mathrm{hv}\;\to {\;\mathrm{g-C}}_{3}N_{4}\;\left(e^{-}{+h}^{+}\right)$$

(2) $$H_{2}{O+h}^{+}\;\to \;\cdot {\mathrm{OH}+H}^{+}$$

(3) $${6H}_{2}{O+4h}^{+}\to {\;4H}^{+}{+O}_{2}↑\;$$

(4) $$O_{2}{+2e}^{-}\;\to \;\cdot O_{2}^{-}\;\;\;\;\;$$

(5) $$h^{+}/\cdot O_{2}^{-}/\cdot \mathrm{OH}+\mathrm{Rhb}/\mathrm{MO}\;\to \;\mathrm{degradation}\;\mathrm{products}\;\;$$

#### 2.4.1. Detection of Zeta Potential

As shown in Table 4, the mechanism of the two synergistic systems was further explored by measuring their zeta potential. g-C_3_N_4_ had an initial potential of −14.4 mV, indicating that the surfaces of its particles were mainly negatively charged. When the dye was then added, the negative surface charge appeared to increase in different cases, due to photocatalysis. The potentials of Rhb and MO were −27 mV and −19.6 mV, respectively. In contrast, when H_2_O_2_ was added, the zeta potential of Rhb and MO decreased to −20.3 mV and –12.6 mV, respectively. This is due to the fact that H_2_O_2_ also acts as an electron trapping agent in the synergistic systems, resulting in a decrease in negative surface charge.

Therefore, it can be presumed that H_2_O_2_ will undergo a reduction reaction with e^-^ to form ·OH, as in Equation (6). When Na_2_S_2_O_8_ is added, the zeta potential did not change much, probably because the reaction of Equation (7) does not cause a change in charge [63,64]. As in Equation (8), SO_4_^−^· can also convert Rhb and MO into small molecules. Under the energy excitation of visible light, H_2_O and O_2_ will produce H_2_O_2_, and H_2_O_2_ will produce OH (see Equations (9) and (10)).

(6) $$H_{2}O_{2}{+e}^{-}\;\to \;\cdot {\mathrm{OH}+\mathrm{OH}}^{-}$$

(7) $$H^{+}{+S}_{2}O_{8}^{2-}\;\to {\;2\mathrm{SO}}_{4}^{-}\cdot {+H}^{+}\;$$

(8) $${\mathrm{SO}}_{4}^{-}\cdot +\mathrm{Rhb}/\mathrm{MO}\;\to \;\mathrm{degradation}\;\mathrm{products}$$

(9) $$O_{2}{+2H}_{2}O+\mathrm{hv}\;\to {\;H}_{2}O_{2}$$

(10) $$H_{2}O_{2}+\mathrm{hv}\;\to \;\cdot \mathrm{OH}$$

From the above reactions, it can be seen that holes (h^+^), superoxide radicals (·O_2_^−^), hydroxyl radicals (·OH), and sulfate radicals (SO_4_^−^·) can degrade rhodamine b and methyl orange solutions.

#### 2.4.2. Free Radical Capture Experiments

In order to determine the main active substances in the photocatalytic degradation of the cationic dye Rhb and anionic dye MO within different synergistic systems, quenching experiments of holes (h^+^) and radicals were carried out, and a blank control was made (see Table 5). Methanol (CH_3_OH), p-benzoquinone (C_6_H_4_O_2_), diphenylamine (C_12_H_11_N), and ethanol (CH_3_CH_2_OH) were chosen as quenching agents for h^+^, ·O_2_^−^, ·OH, and SO_4_^−^·, respectively [65,66]. The quenching reaction times were 90 min for all the Rhb solutions. The time for the quenching reaction of the MO solution was 150 min for the H_2_O_2_ synergistic system and 210 min for the Na_2_S_2_O_8_ synergistic system.

**Figure 8:**
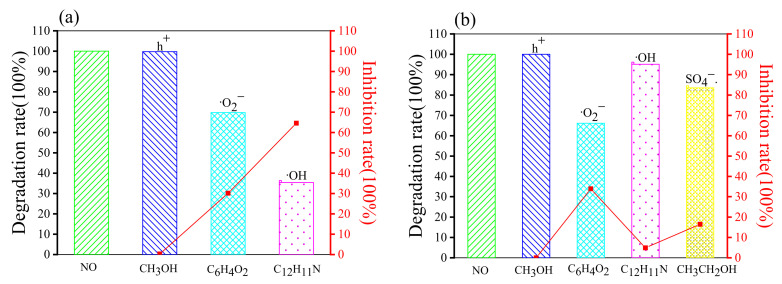
Photocatalytic degradation of Rhb in the synergistic systems with different quenching agents: (**a**) Quenching of Rhb degradation in the H_2_O_2_ synergistic system; (**b**) Quenching of Rhb degradation in the Na_2_S_2_O_8_ synergistic system.

As can be seen in Figure 8a, the degradation of Rhb was 100% in the absence of any quenching agent, and the addition of methanol had almost no effect on the degradation of Rhb solution (showing an inhibition rate of 0.25%). The addition of both p-benzoquinone and diphenylamine had an inhibitory effect on the reaction (the inhibition rates were 30.15% and 64.6%, respectively). The degradation rate decreased from 100% to 35.4% with the addition of diphenylamine, which was 34.45% higher than that of p-benzoquinone, indicating that ·OH was the main active substance within this synergistic system. In Figure 8b, the addition of methanol hardly affected the degradation rate of Rhb. The addition of p-benzoquinone, diphenylamine, and ethanol all significantly inhibited the degradation of Rhb. The inhibition rate of diphenylamine was 4.88%, yet p-benzoquinone had the most prominent effect with 33.88% inhibition, which is about twice as much as ethanol (with an inhibition rate of 16.47%), indicating that ·O_2_^−^ is the main reactive radical.

**Figure 9:**
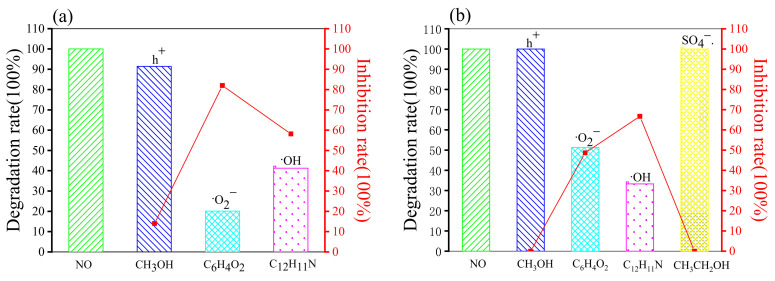
Photocatalytic degradation of MO in the synergistic systems with different quenching agents: (**a**) Quenching of MO degradation in the H_2_O_2_ synergistic system; (**b**) Quenching of MO degradation in the Na_2_S_2_O_8_ synergistic system.

In Figure 9a, the addition of methanol inhibited 8.59% of the MO degradation. The degradation of MO decreased from 100% to 20.08%, and 41.26% after the addition of p-benzoquinone and diphenylamine, respectively, indicating that ·O_2_^−^ and ·OH are the main active substances in the H_2_O_2_ synergistic g-C_3_N_4_ system. The inhibition rate of benzoquinone (79.92%) was 21.18% higher than that of diphenylamine (an inhibition rate of 58.74%), such that ·O_2_^−^ was the more dominant reactive radical in this system. In Figure 9b, the addition of methanol and ethanol did not have much effect on the degradation of MO (both inhibition rates were 0). In addition, the degradation of MO was 48.65% and 66.73% after quenching with ·O_2_^−^ and ·OH, respectively. ·OH was the more dominant reactive radical in this system.

As suggested by the above results, the main reactive substances of anionic and cationic dyes in different synergistic systems are exactly opposite (see Table 6). For the cationic dye Rhb, ·OH and ·O_2_^−^ are the most dominant reactive substances in the H_2_O_2_ and Na_2_S_2_O_8_ synergistic g-C_3_N_4_ photocatalytic degradation systems, respectively. In contrast, in the anionic dye MO, ·O_2_^−^ was the most dominant reactive radical in the H_2_O_2_ synergistic system, and ·OH was the most dominant reactive substance in the Na_2_S_2_O_8_ synergistic system. Thus, it is speculated that this phenomenon is related to contrasting pH level between the anionic and cationic dyes, which are acidic and alkaline, respectively. The mechanism for this is to be further investigated.

#### 2.4.3. Stability Testing

**Figure 10:**
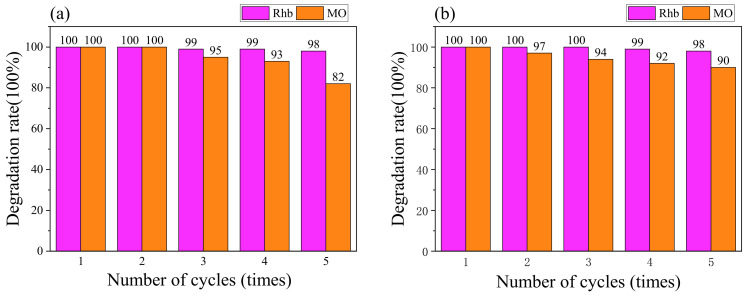
The reusability of collaborative system:(**a**) g-C_3_N_4_/H_2_O_2_ (**b**) g-C_3_N_4_/Na_2_S_2_O_8_.

**Figure 11:**
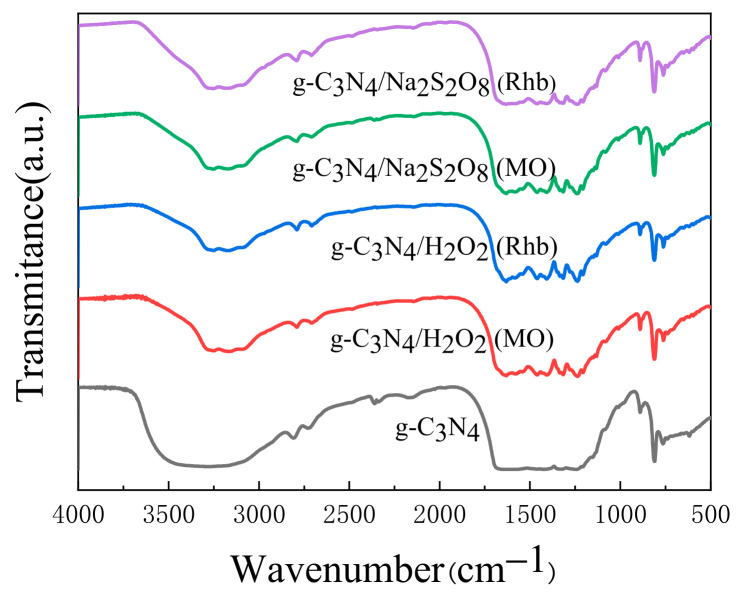
The FT-IR of synergetic system before and after degradation.

The stability and recyclability of the photocatalyst are also important properties for its practical application. In order to determine the stability of the synergistic photocatalytic reaction, repeated experiments were carried out, as shown in Figure 10a. After five cycles, the degradation rate of Rhb in the g-C_3_N_4_/H_2_O_2_ synergistic system only showed a very slight activity decrease, remaining above 98%. By contrast, the MO degradation rate decreased, and the degradation rate was 82% in the 5th cycle, but the first four cycles were above 93%. As shown in Figure 10b, the degradation rates of Rhb and MO in the g-C_3_N_4_/Na_2_S_2_O_8_ synergistic system did not change much and remained above 90% after five cycles. To further investigate the structure and chemical stability of the photocatalyst, FTIR analysis was performed on the samples before and after the photocatalytic reaction. As shown in Figure 11, the FTIR spectra before and after the reaction were almost the same. This indicates that the g-C_3_N_4_ in the synergistic system not only has good stability during the degradation of Rhb and MO, but also has a good reusability performance. In addition, the slight decrease in photocatalytic activity is caused by the mass loss of the photocatalyst during the cycling process.

#### 2.4.4. Principle of Photocatalysis

**Figure 12:**
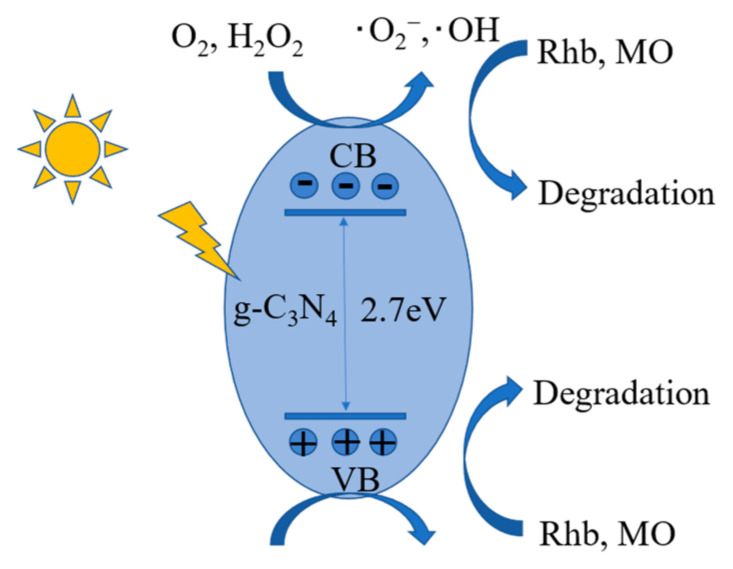
Schematic diagram of the mechanism of synergistic photocatalysis.

As shown in Figure 12, based on the preliminary experimental tests and theoretical analysis, we hypothesized the mechanism of g-C_3_N_4_ synergistic multisystem photocatalytic degradation of anionic and cationic dyes (Rhb and MO). Under the irradiation of visible light, the electrons on the valence band (VB) of g-C_3_N_4_ will be excited to the conduction band (CB), producing photogenerated electrons and holes, i.e., e^−^ and h^+^. The presence of two oxidants, Na_2_S_2_O_8_ and H_2_O_2_, will reduce the complex rate of e^-^ and h^+^, so that more photogenerated carriers will be involved in the degradation of Rhb and MO. h^+^, ·O_2_^−^, ·OH, SO_4_^−^· can all degrade rhodamine b and methyl orange solutions. However, in synergistic systems, the main reactive radicals for the degradation of anionic dyes are ·OH and ·O_2_^−^. The reduction reaction of O_2_ and H_2_O_2_ produces ·OH and ·O_2_^−^; the oxidation reaction of H_2_O produces ·OH, and then the anionic dyes (Rhb and MO) are degraded to small molecules by the ·OH and ·O_2_^−^.

## 3. Experimental Part

### 3.1. Reagents and Apparatus

Experimental reagents: urea, potassium bromide (Tianjin Damao Chemical Reagent Factory, Tianjin, China); rhodamine b (Rhb) (Tianjin Damao Chemical Reagent Factory, Tianjin, China); methyl orange (MO) (Tianjin Guangfu Technology Development Co., Ltd. Tianjin, China); 30% hydrogen peroxide (30% H_2_O_2_), and sodium persulfate (Na_2_S_2_O_8_) (Sinopharm Chemical Reagent Co., Ltd. Shanghai, China); p-benzoquinone (Shanghai Maclean Biochemical Technology Co. Ltd. Shanghai, China); anhydrous ethanol (Tianjin Zhiyuan Chemical Reagent Co., Ltd. Tianjin, China). All the above drugs were analytically pure (AR), and the experimental water was deionized water.

Experimental instruments: optical dark box (GXAS345); ultrasonic cleaner (KQ-100B); muffle furnace and UV–Vis spectrophotometer (UV-26001); Nicolet 380 FTIR spectrometer; Regulus 8100 scanning electron microscope (SEM); X-ray photoelectron spectrometer (XRD).

### 3.2. Preparation of Catalyst

Weigh a certain amount of urea, wrap it with tinfoil, place it in a muffle furnace and heat it up to 520 °C, hold it for 2 h, then heat it up to 550 °C and hold it for 2 h, wait for it to cool down to room temperature, transfer the roasted light yellow solid to a mortar and grind it to get g-C_3_N_4_.

### 3.3. Structural Characterization and Performance Testing

#### 3.3.1. Structural Characterization

Infrared spectroscopy was conducted using Fourier transform infrared spectroscopy (FT-IR): samples and pure KBr were dried and ground to a particle size < 2 µm, then pressed into translucent sheets for measurement (scanning range 4000–400 cm^−1^).

Crystal structure of samples using X-ray diffraction (XRD): scanning range 5–90 °C; scanning rate 2 °C/min; step size 0.02; current 40 mV; voltage 40 kV.

The microstructure of the sample was analyzed using scanning electron microscopy (SEM): the sample was attached to a black conductive adhesive, and measured after vacuum gold spraying with an acceleration voltage of 1.0 kV.

#### 3.3.2. Photocatalytic Performance Testing

Rhb solutions were prepared at mass concentrations of 5, 10, 15, 20, 25, 30, 35, 40, 45, 50 mgL^−1^. The absorbance (A) of each solution was measured using an UV–Vis spectrophotometer at 550 nm, and the equation of the standard curve of absorbance and mass concentration of Rhb (ρ_1_, mgL^−1^) was recorded as A = 0.0317ρ_1_ + 0.0012, R^2^ = 0.9996. Preparation of MO solutions at mass concentrations of 1, 2, 5, 10, 15, 20, 25, 30 mgL^−1^. The absorbance (B) of each solution was measured using an UV–Vis spectrophotometer at 465 nm, and the equation of the standard curve of absorbance and mass concentration of MO (ρ_2_, mgL^−1^) was recorded as B = 0.0489 ρ_2_ + 0.0048, R^2^ = 0.9997.

A xenon lamp was used to simulate solar light conditions, and Rhb (150 mL, 50 mg·L^−1^) and MO (100 mL, 20 mg·L^−1^) were added as the target pollutants, and 100 mg and 50 mg of g-C_3_N_4_ were added through their respective dark reactions for 60 min, and then appropriate amounts of 30% H_2_O_2_ solution and 0.1 mol·L^−1^ Na_2_S_2_O_8_ solution were added, respectively. The light source was turned on for the photocatalytic experiments, and samples were taken at different intervals, filtered through a 0.45 µm filter membrane, and then measured using an UV spectrophotometer. The photocatalytic and synergistic system for the degradation of anionic dyes are shown in Equations (11) and (12);

(11) $$-\ln\;\left({C/C}_{0}\right)=\mathrm{kt}$$

(12) $$\varphi /100=\Delta {C/C}_{0}{\;\times \;100=[(C}_{0}-{C)/C}_{0}]\;\times \;100$$

wherein “C” is the mass concentration of TC at time t, in mg/L; “C_0_” is the mass concentration of initial TC, in mg/L; “φ” is the degradation rate (or adsorption rate), in %; “∆C” is the mass concentration of TC reduction after time t, in mg/L; “k” is the reaction rate constant, in min^−1^; and “t” is the reaction time, in min.

## 4. Conclusions

Compared with the pure g-C_3_N_4_, the degradation rates of MO and Rhb were increased by 2.5 and 3.5 times for the above two synergistic systems, respectively. Within 60 min of photocatalytic reaction, the degradation rates of MO and Rhb were 90.71% and 98.54% in the H_2_O_2_ synergistic system, respectively, and 92.61% and 99.56% in the Na_2_S_2_O_8_ synergistic system, respectively. It can be seen that the addition of an oxidant is beneficial to the effective separation and migration of photogenerated electron-hole pairs. The results of Na_2_S_2_O_8_ and H_2_O_2_ concentration on the degradation of pollutants showed that the photocatalytic degradation of the synergistic system showed a tendency to become better with the increase in the drop volume. Zeta potential and radical quenching experiments showed that ·OH and ·O_2_^−^ were the main reactive radicals for the degradation of dyes in these synergistic systems. The experimental phenomena suggest that there are distinct pattern difference in the main reactive substances of anionic and cationic dyes in different synergistic systems, which need to be further investigated.

## Figures and Tables

**Table 1:** Degradation rate of Rhb in synergistic systems.

Time (min)	Degradation Rate of g-C_3_N_4_/H_2_O_2_ (100%)	Degradation Rate of g-C_3_N_4_/Na_2_S_2_O_8_ (100%)
−60	0	0
0	1.84	1.84
10	43.57	13.81
20	64.60	79.92
30	80.94	97.66
40	87.14	98.73
50	93.35	99.24
60	98.54	99.56
90	100	100

**Table 2:** Degradation rate of MO in synergistic systems.

Time (min)	Degradation Rate of g-C_3_N_4_/H_2_O_2_ (100%)	Degradation Rate of g-C_3_N_4_/Na_2_S_2_O_8_ (100%)
−60	0	0
0	15.18	15.18
10	31.87	44.96
20	52.75	65.73
30	68.33	86.51
60	90.71	92.61
90	99.90	98.70
150	100	99.80
210	100	100

**Table 3:** Comparison of the various parameters of dye degradation.

Catalyst	Quality (mg)	Contaminants	Concentration (mg/L)	Volume (mL)	Time (h)	Degradation Rate (100%)	Speed (min^−1^)
ZHS/ZTO	50	MO	10	200	2	68.45	-
Sulfur/chlorine/g-C_3_N_4_	50	Rhb	10	50	-	-	0.01683
CeTiO_4_/g-C_3_N_4_	100	Rhb	10	100	2.4	95.7	0.0202
Chlorine/g-C_3_N_4_	50	Rhb	10	100	-	-	0.049
TiO_2_@SiO_2_	100	Rhb	20	100	12	86	-
TiO_2_@SiO_2_	100	MO	20	100	12	38	-
{[Ag_2_(mu-NO_3_) L1]} n	10	Rhb	49.6	20	6	85.2	0.00747
{[Ag_2_(mu-NO_3_) L1]} n	10	MO	18.68	20	6	70.6	0.00354
This study (g-C_3_N_4_)	100	Rhb	50	150	1.5	100	0.06907 0.10511
This study (g-C_3_N_4_)	50	MO	20	100	2.5 3.5	100	0.07753 0.03978

**Table 4:** Zeta potential of g-C_3_N_4_ material.

Materials	Zeta Potential (mV)	Materials	Zeta Potential (mV)
g-C_3_N_4_	−14.4	g-C_3_N_4_ (MO)	−19.6
g-C_3_N_4_ (Rhb)	−27	g-C_3_N_4_ (MO, H_2_O_2_)	−12.6
g-C_3_N_4_ (Rhb, H_2_O_2_)	−20.3	g-C_3_N_4_ (MO, Na_2_S_2_O_8_)	−29.1
g-C_3_N_4_ (Rhb, Na_2_S_2_O_8_)	−27.5		

**Table 5:** Free radical capture control situation.

	Degradation Rate (100%)					Inhibition Rate (100%)				
	No	h^+^	·O_2_^−^	·OH	SO_4_^−^·	No	h^+^	·O_2_^−^	·OH	SO_4_^−^·
Rhb (g-C_3_N_4_/H_2_O_2_)	100	99.75	69.85	35.4	-	0	0.25	30.15	64.6	-
Rhb (g-C_3_N_4_/Na_2_S_2_O_8_)	100	100	66.12	95.12	83.53	0	0	33.88	4.88	16.47
MO (g-C_3_N_4_/H_2_O_2_)	100	91.41	20.08	41.26	-	0	8.59	79.92	58.74	-
MO (g-C_3_N_4_/Na_2_S_2_O_8_)	100	100	51.35	33.27	100	0	0	48.65	66.73	0

**Table 6:** Main reactive radicals of different synergistic systems.

Type	Target Pollutant	Synergistic Multisystem	Main Active Radical
cationic dyes (alkaline dye)	Rhb	H_2_O_2_	·OH
		Na_2_S_2_O_8_	·O_2_^−^
anionic dyes (acid dyes)	MO	H_2_O_2_	·O_2_^−^
		Na_2_S_2_O_8_	·OH

## Data Availability

Not applicable.

## Sample Availability

Samples of the compounds are available from the authors.
